# Systemic bias in medical consensus: how flawed panels and lack of evidence can distort definitions and guidelines: the case of Vogt-Koyanagi-Harada disease: **Perspective**

**DOI:** 10.1186/s12348-026-00617-7

**Published:** 2026-07-09

**Authors:** Carl P. Herbort, Ioannis Papasavvas, Ahmed M. Abu El-Asrar, Abdulrahman F. AlBloushi, De-Kuang Hwang, Vania Lages, Wen-Jung Lo, Kazuich Maruyama, Piergiorgio Neri, Masaru Takeuchi, Yoshihiko Usui

**Affiliations:** 1 Retinal and Inflammatory Eye Diseases, Centre for Ophthalmic Specialised Care (COS), Lausanne, Switzerland; 2 Department of Ophthalmology, Arab National Bank Chair in Ophthalmology, College of Medicine, King Saud University, Riyadh, Saudi Arabia; 3 Department of Ophthalmology, College of Medicine, King Saud University, Riyadh, Saudi Arabia; 4 Department of Ophthalmology, Taipei Veterans General Hospital, Taipei, Taiwan; 5 Department of Ophthalmology, School of Medicine, National Yang Ming Chiao Tung University, Taipei, Taiwan; 6 Unidade Local de Saúde Alto Ave, Guimarães, Portugal; 7 Department of Ophthalmology, Taichung Veterans General Hospital, Taichung, Taiwan; 8 Department of Vision Informatics, Graduate School of Medicine, Osaka University, Osaka, Japan; 9 Integrated Surgical Institute, The Eye Department, Cleveland Clinic Abu Dhabi, Abu Dhabi, UAE; 10 Cleveland Clinic Lerner College of Medicine, Case Western Reserve University, Cleveland-Ohio, USA; 11 Department of Ophthalmology, National Defence Medical College, Tokorozawa, Saitama Japan; 12 Department of Ophthalmology, Tokyo Medical University, Tokyo, Japan

## Abstract

**Background:**

The creation of diagnostic criteria for a disease is always challenging in medicine. Consensus meetings and expert’s opinions are sometimes responsible for establishing useful and precise diagnostic criteria but, unfortunately, not all the consensus meetings are based on evidence-based conclusions.

**Methods:**

A perspective analysis of the historical evolution of diagnostic criteria for Vogt-Koyanagi-Harada.

**Results:**

We examine the historical evolution of diagnostic criteria for Vogt–Koyanagi–Harada disease, an autoimmune condition targeting melanocyte-containing tissues, primarily affecting the choroid and potentially involving the skin, ears, and meninges. Early diagnostic criteria proposed in the late twentieth century were considered inadequate, leading to an international consensus conference in 1999 and the publication of revised diagnostic criteria in 2001. However, these criteria contained major conceptual flaws, notably the combination of acute and chronic clinical features that rarely occur simultaneously. In addition, important diagnostic tools such as indocyanine green angiography were largely excluded due to prevailing local practices and skepticism regarding their use. These limitations hindered accurate diagnosis and delayed recognition of VKH as a disease with distinct acute-onset and chronic forms requiring different diagnostic frameworks and management strategies. Subsequent studies corrected some deficiencies but often generated complex criteria difficult to apply in routine practice.

**Conclusion:**

We would like to emphasize the risks of poorly structured consensus processes and propose safeguards to ensure scientifically rigorous and clinically useful recommendations.

## Intellectual conformism and social rationality

Samuel Fitoussi in his book “Why do intellectuals go wrong” explains the shifts of intellectual thought [1]. He argues that intellectuals can profess misconceptions without damaging their reputation as long as such statements are in agreement with the general trend of thought of the moment, with what he calls the social rationality. One important reason for erroneous intellectual interpretation of things is conformism. In the appraisal of phenomena, two different rationalities clash with each other; on one side, epistemic rationality meaning the rationality that admits valid explanation of innovative concepts, and on the other side, social rationality, meaning rationality that aligns with the generally accepted, though possibly wrong explanation of things, in other words conformism.

He cites the example of Copernicus and Galileo Galilei who posited that the sun, not the earth, was at the centre of the universe (heliocentric theory). Copernicus and Galilei defended the epistemic rationality, the rationality able to admit the valid explanation of things. However, this was not, at the time, the dominant social rationality or the generally accepted explanation that prevailed. The price to pay by standing with the epistemic rationality against the social rationality is very high and Galilei had to officially withdraw his theory while Copernicus’ book published in 1543 remained in the index of prohibited books by the catholic church until 1758. On the other hand, it is much more rewarding socially or professionally to adopt the dominant social rationality following consensus rules prevailing in a given era and area even if their validity may be doubtful, because by doing so, a given person or professional will score points and will be well considered socially. Thus, conformism is much more comfortable, and this largely explains why intellectuals can go wrong explains Fitoussi [1]. 

## Medical consensus and the temptation of conformism

There is no reason to believe that things are different in medicine. Opinion leaders yearn to be well considered and therefore have a natural tendency to seek conformism. One way to influence medical debate is through the organisation of consensus meetings, which often privilege generally accepted concepts via pre-structured proceedings by panels that are presented to an international floor, thus institutionalising medical definitions.

In the field of ophthalmology in which we work, uveitis, several examples of conformism-biased consensus meetings and/or opinion decisions can be cited, namely (1) consensus diagnostic decisions on HLA-A29 Birdshot Retinochoroiditis, a disease involving the choroid and (2) the white dot terminology including unrelated diseases grouped together merely because of their similar clinical appearance [2, 3]. Consensus processes bear with them a potential harmfulness resulting in the validation of concepts often biased to conform to the prevailing social rationality. In the two cited examples it took years and even decades to rectify these consensus or opinion derived concepts [4, 5]. One such example is the definition of the diagnostic criteria of VKH through a consensus conference in 1999 and published in 2001 [6]. We take advantage of this case to demonstrate the slow trial and error process to reach a valid definition of diagnostic criteria of a disease.

## Historical and epidemiological context of VKH

Vogt-Koyanagi-Harada disease (VKH) is an autoimmune disease directed against melanocytes, usually starting in the choroidal stroma and subsequently also involving melanocytes in the anterior segment of the eye and in other tissues such as the ear, skin and meninges. The disease is more prevalent in more darkly pigmented races such as Indo-Asiatic, native American, Hispanic and Middle Eastern populations and less so among Caucasians and sub-Saharan Africans [7, 8]. 

Japan is one of the countries where the prevalence of VKH has always been high. A recent epidemiological study showed that today VKH is still the second most frequent diagnosis among Japanese uveitis patients [9]. Moreover, among the countries with high VKH prevalence, Japan had one of the most developed medical systems at the beginning of the 20th century. Indeed, since the Japanese restoration of power from the Shogun to the Emperor in 1868 and the western orientation of the organization of the state, medical education was imported from Germany that was then the leading country in medical sciences. It is therefore not astonishing that the disease was described and characterized in the literature by Japanese doctors. Many articles on VKH by Japanese authors were published in German medical journals including the groundbreaking article by Yoshizo Koyanagi published in German in 1929 [10]. Einosuke Harada published his article on the posterior involvement of VKH in 1926 in a Japanese journal [11], so completing the eponym after the first mention of the disease by Alfred Vogt in Basel, Switzerland [12]. 

## The importance of early diagnosis of VKH

**Fig. 1:**
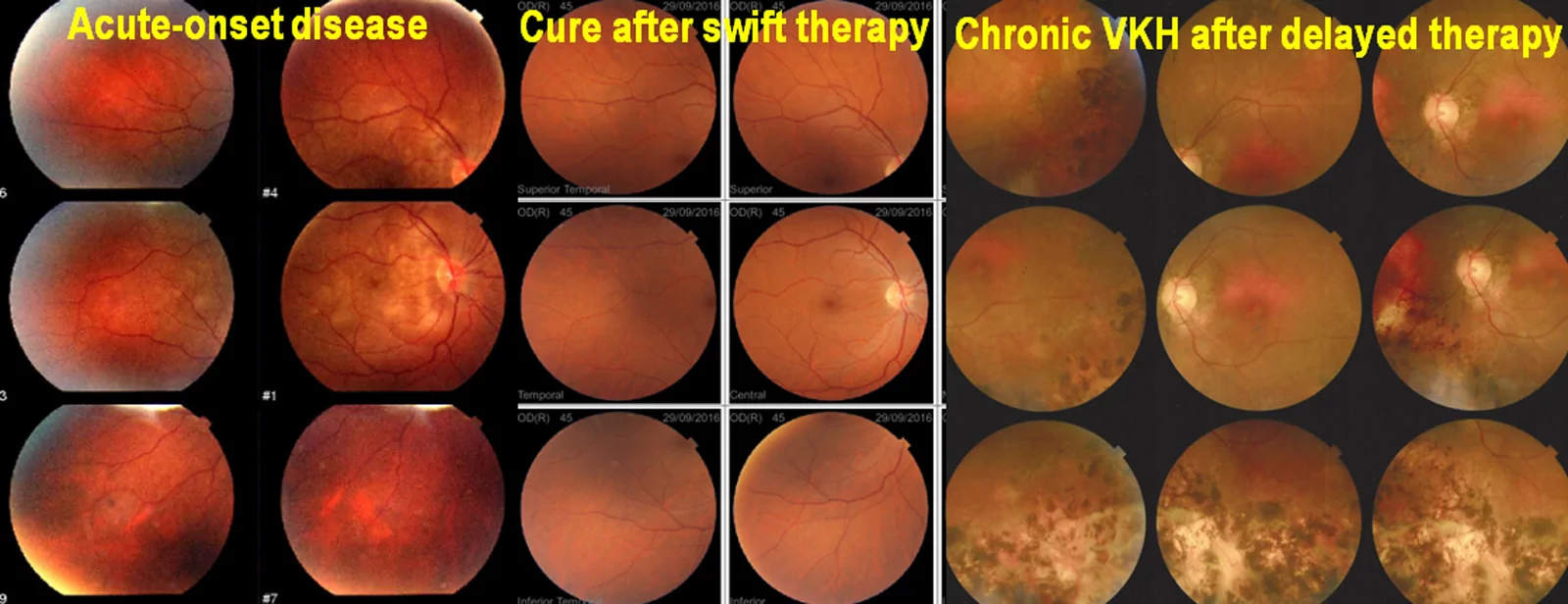
Importance of immunosuppressive treatment within the therapeutic window of opportunity**.** Left sextet of fundus figures showing early-onset disease that was treated promptly within two weeks of start of symptoms with steroidal and non-steroidal immunosuppression during 40 months resulting in the cure of disease with a follow-up without treatment of 6 years (middle sextet of figures). The right set of 9 fundus figures shows case with delayed diagnosis and treatment evolving chronically despite massive treatment with numerous chorioretinal scars

If not diagnosed early and not treated promptly with appropriate therapeutic intervention, the disease will progress to chronic recurrent evolution. Treatment will then have to be aggressive and prolonged and the evolution of such chronic disease can be deleterious associated with many complications [13]. **(**Fig. 1**)** Therefore, prompt diagnosis of the disease is of crucial importance and hence the need for robust diagnostic criteria.

## Early diagnostic attempts and the 1999 consensus meeting

Logically the first set of diagnostic criteria for VKH were also stated by a Japanese clinician, Dr. Sugiura, first in 1976 in Japanese [14] and in 1978 in English in the Japanese Journal of Ophthalmology [15]. In 1980, the diagnostic criteria of the American Society of Uveitis were published [16]. 

Both these two sets of criteria were judged as being unsatisfactory and because of their poor performance two university institutions in Los Angeles, California decided to organise a consensus meeting at Lake Arrowhead in the San Bernardino Mountains and to call in international colleagues to establish “revised diagnostic criteria for the diagnosis of VKH” which were published in 2001 [6]. 

When this consensus meeting was held, ocular imaging, with the availability of indocyanine green angiography (ICGA) since 1992 had made significant progress in the analysis of the choroidal space including the choroidal stroma the primary exclusive development of VKH lesions before their spill-over into other ocular structures [17, 18, 19, 20]. Despite the fact that ICGA had been developed in New York by the group of LA Yannuzzi at the Manhattan Eye and Ear Infirmary [17, 18], the US doctors and institutions remained little motivated to use ICGA and Yannuzzi himself expressed his incomprehension of this situation [21]. 

Thus, the consensus meeting was held in a setting with a “social rationality” corresponding to the local context including, among others, refractoriness to ICGA and negative positions towards cerebrospinal fluid (CSF) analysis because of fear of potential medico-legal consequences of this invasive procedure. Thus, the influence of the local positions was not negligible as the plan of the proceedings was put together by a mainly local steering committee. Moreover, 42% of participants were from the organising country which has a relatively low VKH prevalence (0.9–1.3%). While only 16% of participants were Japanese, despite VKH accounting for 8.1–13% of uveitis cases in Japan with the respective expertise this implies. [9, 22–25]

During the proceedings there was a strong pressure to exclude lumbar puncture CSF analysis, which today, with the new imaging possibilities of the choroid available, using Optical Coherence Tomograph (OCT) and ICGA, appears probably justified. It was, however, unnatural to exclude ICGA from the debate, knowing its crucial role in the assessment of stromal choroiditis entities such as VKH [26]. 

## Flaws in the revised VKH criteria

**Fig. 2:**
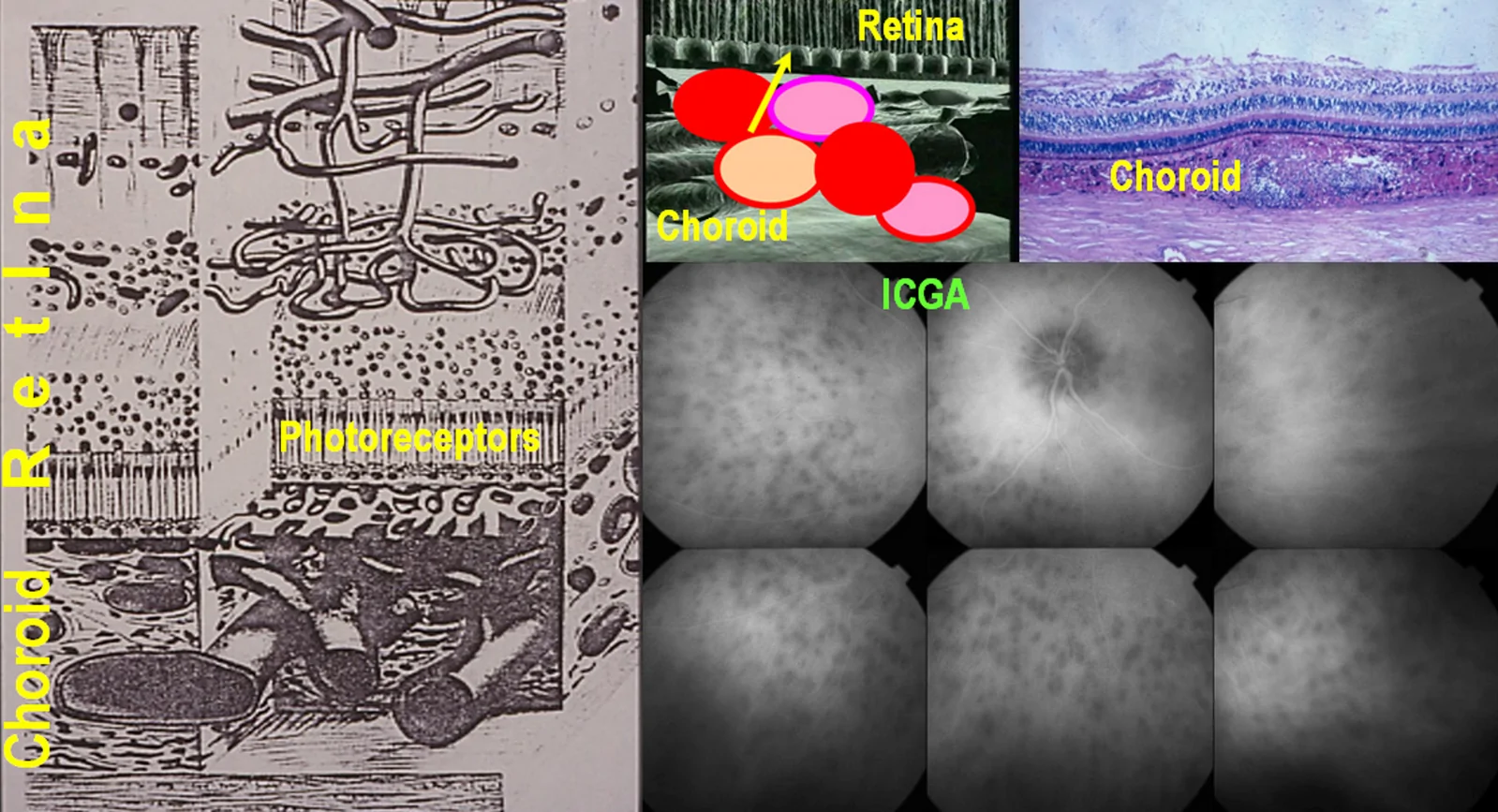
Use of ICGA for rapid diagnosis of VKH. VKH starts with infiltration of the choroid, the vascularised space under the retina. Top right, histologic section showing inflammatory foci infiltrating and thickening the choroidal space as schematised by cartoon (top middle) showing the process of infiltration of the choroid before inflammation spills over into the retina. Bottom right, ICGA showing occult choroidal foci visible before the disease is clinically apparent allowing early diagnosis and early treatment

The most critical flaw of the 1999 revised diagnostic criteria was that they mixed acute and chronic signs, clinical features that do not occur simultaneously, which is not an opinion but a verifiable fact. This flaw had already plagued the Sugiura and the American Uveitis Society criteria. The consensus group created three categories: complete, incomplete and probable VKH. Yet the “complete” form was rarely seen in practice due to the non-overlapping nature of acute and chronic features. Many articles were written following the publication of this consensus meeting with limited relevance, analysing VKH series along these principles and it became quickly apparent that the complete form could almost never be diagnosed, precisely because acute and chronic signs do not occur simultaneously. Not only were these definitions useless and contributed nothing to improve the practical appraisal and management of VKH [27], but they also additionally hampered progress by delaying the emergence of the crucial concept that VKH should be subdivided into acute-onset and chronically evolving forms [28], with diagnostic criteria applied separately to one and the other of these two forms [29, 30]. Furthermore, including ICGA in the elaboration of the diagnostic criteria should have been considered as it precisely and swiftly accounts for choroidal stromal inflammation and was practiced routinely in Europe and Japan at the time [19, 20, 31, 32]. **(**Fig. 2**)** In summary, the Sugiura, the AUS and the revised criteria did not incorporate objective and quantifiable imaging elements either because they were not available or because they were not taken into account. So, all 3 sets of criteria were strictly opinion-based in the way clinical signs were used for classification.

## Later adjustments and lingering issues

Subsequent consensus efforts corrected some flaws, finally introducing separate criteria for acute and chronic VKH. However, ICGA and Enhanced Depth Imaging OCT (EDI-OCT) were still excluded, despite their central role in assessing choroidal inflammation [33]. Moreover, no precise timeline was given to distinguish acute from chronic forms.

Oddly the same consensus group included ICGA in their classification criteria of HLA-A29 Birdshot retinochoroiditis, a stromal choroiditis absolutely comparable to VKH [34]. The authors of these subsequent consensus decisions refrained from speaking of diagnostic criteria and put an accent on the scientific aspect of their process, calling it classification criteria. It is however very well known that readers will tend to consider them as diagnostic criteria.

In 2018, Yang et al. published the most precise diagnostic data relying on 634 Chinese VKH patients that were analysed with rigorous statistical methods [35]. The diagnostic criteria appropriately distinguished acute-onset and chronic diseases and included relevant imaging methods such as EDI-OCT. Although scientifically impeccable, the very elaborate list of diagnostic criteria and variants made the criteria very complicated for practical use. Additionally, the system did not include the use of ICGA nor was a precise time-line given delineating chronic from acute-onset cases.

## Towards practical evidence-based criteria

Beside the necessary statistical determination of disease characteristics performed by groups using machine learning [33, 34], the aim to determine practical clinically useful diagnostic criteria that was attempted by the consensus work performed by the “revised diagnostic criteria of VKH” was a commendable and necessary step as long as safeguards were respected such as avoiding biases linked to the promoters positions and integrating evidence-based criteria when available such as precise measurement methods of choroidal inflammation rather than consensus-based criteria. These conditions were however not fulfilled.

We have been working on generating such practical, clinically useful diagnostic criteria [29]. 

**Fig. 3:**
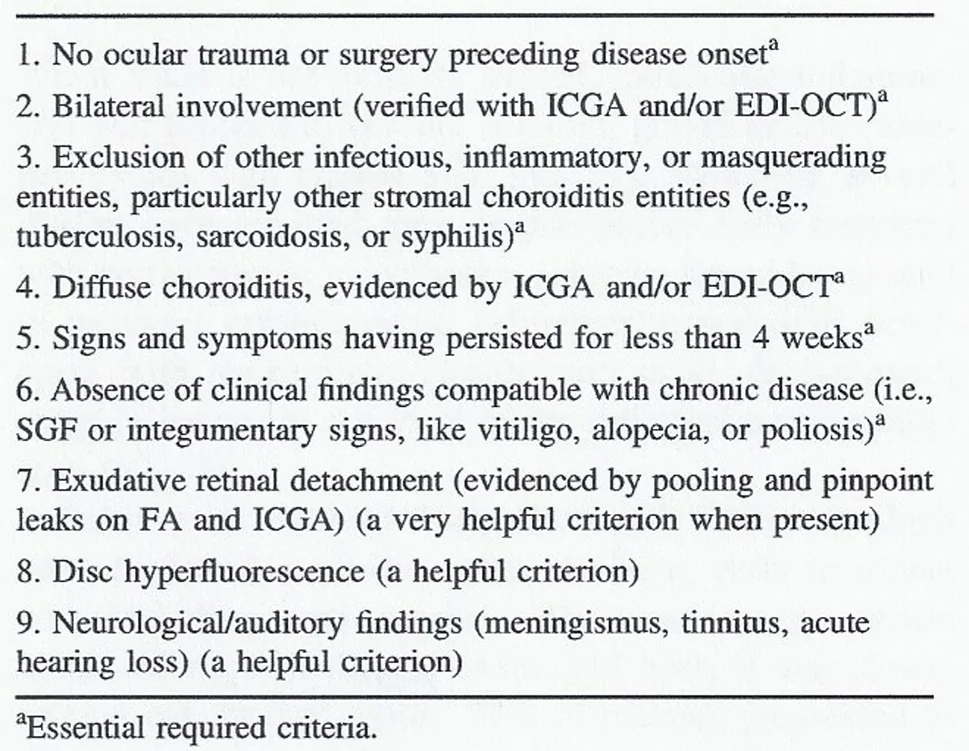
**Diagnostic criteria of acute-onset VKH**

Reprinted from Herbort CP Jr, et al. [29]

**Fig. 4:**
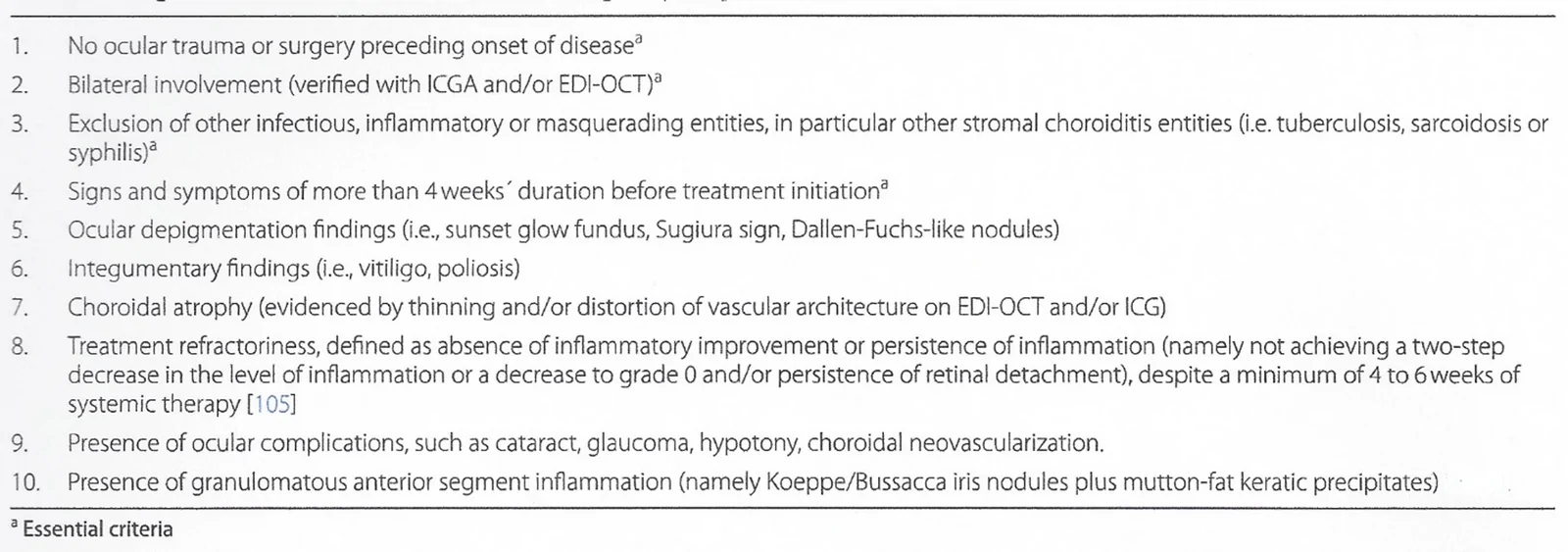
Diagnostic criteria of chronically evolving VKH

Reprinted from Urzua, et al. [30]

(1) Acute-onset and chronic forms were separated with each having their specific criteria [29, 30]. **(**Figs. 3 and 4**)**

(2) The precise time course delineating the two forms was precisely determined and was shown to be three, possibly four weeks after the onset of symptoms, based on the published timespan determined to be within the therapeutic window of opportunity defined as a time interval following initial-onset disease during which adequate treatment will substantially modify the disease outcome and possibly even lead to cure, similar to what has been described for rheumatoid arthritis [36]. An alternate definition of the time-limit of the therapeutic window of opportunity in early-onset disease was determined as the disease phase preceding anterior segment inflammation. Although patients rarely present so early, if effective treatment is initiated during this phase the proportion of chronically evolving cases is minimal and the proportion of cured cases is high [37]. 

(3) Finally, we included relevant up-to-date investigational imaging methods [19, 20, 31, 32, 38]. 

The presented criteria were based, newly, on a combination of clinical elements and more recent imaging investigations crucial for VKH as well as the strict separation of acute and chronic signs. As for the “revised diagnostic criteria”, they are open for testing in VKH cohorts, although this was not the primary purpose of our approach more turned towards generating a practical clinical diagnostic framework. Indeed, testing may be warranted and it was testing of the “revised diagnostic criteria” that unveiled their inadequacy failing to identify the complete form in many studies, citing here only a few [27, 39, 40, 41]. 

## **Adverse consequences of inappropriate consensus decisions**

One of the collateral damages of inadequate consensus decisions validated by an international group as was the case for the “revised diagnostic criteria” with limited relevance and practical impact is that they are engraved in marble and infuse the medical literature in textbooks and review articles which will take decades to be rectified, as such concepts will be repeated from one article or chapter to the next. This case highlights the inherent risks of consensus meetings when used to define diagnostic criteria or clinical guidelines. In this instance, a well-intentioned group sought to improve VKH diagnostic criteria. Yet, the process was shaped by the organisers’ preconceived views and dominated by local participants who lacked experience with key diagnostic tools like ICGA. The outcome was not only inadequate but delayed the necessary shift towards viewing VKH as a disease with two distinct clinical forms requiring different diagnostic and management strategies [28]. In the era of evidence-based medicine, reliance on consensus and opinion-based decisions is increasingly hard to justify.

## **Recommendations for a better consensus process**

Nevertheless, this type of approach is still being used presently in our field of uveitis. This is the reason why it is important to draw attention to the pitfalls of such processes. Following the publication coming from one of the main uveitis associations recommending to give up the “White Dot Syndromes” terminology, groups were formed to analyse and determine diagnostic criteria of several of the diseases previously included in this obsolete potpourri group [42]. This article serves as a warning: when panels are inappropriately composed or driven by vested interests, the risk for flawed outcomes is real. As diverse interpretations about these diseases co-exist, the danger is real that such a process is used by a group of clinicians to put forward its prerogatives. We were some colleagues that renounced to take part in this process named the MUV Taskforce because of questionable composition of steering panels. Others had to threaten to withdraw from the group if controversial formulations were not abandoned.

Consensus-based methods should not be rejected outright. However, if estimated to be useful, they must follow strict scientific and ethical safeguards. (1) The steering panel should include diverse recognised experts, including those with dissenting views. The definition of “recognised experts” could rely on a combination of different parameters such as a minimum number of publications, number of patients or years of practice in a high prevalence population for a given disease. (2) Institutions and participants must be varied in background and expertise. (3) Only informed, active or recognised professionals should vote to minimize uninformed decisions. (4) Evidence-based data must support decisions whenever possible. (5) Dissenting opinions must be acknowledged in final documents as consensus is not unanimity [43]. (6) Full methodological transparency should be provided including details on how consensus was reached, also reporting controversial points.

By adhering to such principles, consensus processes can possibly yield rigorous, replicable and scientifically valid recommendations [44]. 

## Data Availability

No datasets were generated or analysed during the current study.
